# On the Road to Camarón: The Sleep of an Ultra-Endurance Athlete Cycling 10,000 km in 24 Days

**DOI:** 10.3390/ijerph19084543

**Published:** 2022-04-09

**Authors:** Mathieu Nédélec, Maxime Chauvineau, Gaël Guilhem

**Affiliations:** Laboratory Sport, Expertise and Performance (EA 7370), French Institute of Sport (INSEP), 11 Avenue du Tremblay, 75012 Paris, France; maxime.chauvineau@insep.fr (M.C.); gael.guilhem@insep.fr (G.G.)

**Keywords:** recovery, performance, stress, master athlete

## Abstract

The impact of sleep on performance is fundamental for ultra-endurance athletes, but studies on this issue are rare. The current investigation examined the sleep and performance of a cyclist engaged in a simulated 10,000 km tour. The sleep behavior of the athlete (age, 57; height, 1.85 m; mass, 81 kg) before, during (i.e., 23 nights), and after the tour was monitored using a reduced-montage dry-electroencephalographic (EEG) device. The daily performance (i.e., number of kms) was recorded throughout the race. The cyclist set a new world record, completing 10,358 km in 24 days with a mean daily distance of ≈432 km in approximately 16 h, i.e., an average speed of ≈27 km/h. Sleep duration throughout the tour (5:13 ± 0:30) was reduced compared to the baseline sleep duration (7:00 ± 1:00), with a very large difference (ES = 2.3). The proportion of N3 during the tour (46 ± 7%) was compared to the measured N3 proportion during the baseline (27 ± 5%) and was found to be systematically outside the intra-individual variability (mean ± 1 SD), with a very large difference (ES = 3.1). This ultra-endurance event had a major influence on sleep-duration reduction and a notable modification in sleep architecture. The increase in slow-wave sleep during the race may be linked to the role of slow-wave sleep in physiological recovery.

## 1. Introduction

Multiday ultra-endurance events present athletes with a significant number of stressors that may include sleep restriction [1] or insufficient energy intake [2]. Lahart et al. [3] monitored the sleep of four cyclists participating in the Race Across America, a 4856 km continuous cycle race they completed in a time of 6 days 10 h and 51 min. Actigraphic results showed that, on average, cyclists slept less than 2.4 h per racing day with impaired sleep efficiency (24 ± 4%), sleep onset latency (83 ± 6 min), and percentage moving time (47 ± 4%), compared with baseline values. Overreached endurance athletes demonstrated a progressive decrease in actual sleep duration, sleep efficiency, and immobile time, during an overload period, suggesting substantial sleep disturbances that may have been related to mild muscle fatigue or soreness resulting from the high training loads [4]. The impact of sleep on ultra-endurance performance is fundamental for ultra-endurance athletes, which may be explained by the role of sleep on recovery [3]. Sleep restriction may be detrimental to the outcome of the recovery process, causing a slowing of muscle damage repair, greater mental fatigue, and higher risk of injury [5,6,7]. There is limited published information on the effect of fragmented sleep on extreme endurance performance [8]. In addition, the high inter-individual sleep variability among athletes [3,9] underlies the importance of a case-study approach [10]. The current investigation examined the sleep and performance of an ultra-endurance cyclist engaged in a simulated 10,000 km tour on a stationary bicycle from Paris (France) to Camarón de Tejeda (Mexico).

## 2. Materials and Methods

### 2.1. Subject and Study Design

The participant visited the laboratory at the French Institute of Sport (Paris, France) before the simulated tour to perform a set of measurements: a 16-day actigraphic recording (CamNtech, MotionWare 8) with a high sleep–wake threshold (>80 activity counts is scored as wake) applied to the data set; a night with a portable complete polysomnography device (Nox A1; Resmed); and a night of familiarization with the reduced-montage dry-electroencephalographic (EEG) device (Dreem headband) [11] used during the tour. The exclusion criteria checked prior to the start of the tour were as follows: (a) an average sleep duration <6 or >9 h per night from Sunday to Thursday; (b) an average lights-out time earlier than 21:00 from Sunday to Thursday; (c) an average wake-up time later than 09:00 from Monday to Friday; (d) daily consumption of alcoholic beverages and/or more than 300 mg of caffeine, and/or the use of antidepressant medications; (e) sleep complaints (i.e., Pittsburgh Sleep Quality Index > 5) [12] and an extreme morning or evening chronotype on the Horne and Ostberg questionnaire (i.e., <31 and >69) [13]; (f) polysomnography-confirmed sleep disorders, such as sleep apnea (apnea–hypopnea index > 10) and other sleep disorders (periodic limb movement syndrome, hypersomnia, insomnia, circadian sleep rhythm disorders, or narcolepsy); and (g) shift worker. The performance achieved on a daily basis (i.e., number of kms) was recorded (Polar V800, Kempele, Finland) for the duration of the race. The participant slept in the same environment throughout the simulated tour, i.e., a camp bed installed close to the bicycle. Electroencephalographic data was collected during two nights after the tour to assess the recovery.

The subject is a 57-year-old ultra-endurance athlete (height, 1.85 m; mass, 81 kg) holding several ultra-endurance world records and world championship titles gleaned during the last twenty years. He was informed about the purpose of the study, and the main investigator answered any questions. This study was undertaken with the agreement of the local ethics committee (East III, France. Ref. 170605) and the recommendations of the Helsinki Declaration.

### 2.2. Procedures

In field-based studies that involve data collection over consecutive nights, activity monitors are typically preferred over polysomnography—the gold standard for monitoring sleep—because they are wearable, are non-invasive, and operate remotely without an attendant technician. In the present study, the subject was equipped with a reduced-montage dry-EEG device (Dreem headband) [11]. The device is a wireless headband worn during sleep, which records, stores, and automatically analyzes physiological data in real time. Following the recording, the device connects to a mobile device via Bluetooth to transfer aggregated metrics to a dedicated mobile application and via Wi-Fi to transfer raw data to the sponsor’s server. Five types of physiological signals are recorded via three types of sensors embedded in the device: (1) brain cortical activity via five EEG dry electrodes yielding seven derivations (FpZ-O1, FpZ-O2, FpZ-F7, F8-F7, F7-O1, F8-O2, FpZ-F8; 250 Hz with a 0.4–35 Hz bandpass filter); (2–4) movements, position, and breathing frequency via a 3D accelerometer located over the head; and (5) heart rate via a red-infrared pulse oximeter located in the frontal band. Previous validation studies have demonstrated the capacity of the device to both monitor sleep-related physiological signals and, then, process them accurately into sleep stages. The automatic sleep-staging classification performed by the device is of similar performance to that of the consensus of five scorers using medical-grade polysomnographic data [11]. The following dependent sleep variables were calculated: time in bed (TIB), the number of minutes from lights-out to lights-on; total sleep time (TST; min), the time spent in any stage of sleep (i.e., light sleep: N1 + N2, slow-wave sleep (SWS; N3), and rapid eye movement (REM) sleep); sleep efficiency (SE; %), TST/TIB × 100; sleep onset latency (SOL; min), the number of minutes from lights-out to the first epoch of any stage of sleep (i.e., N1, N2, SWS, and REM); and wake after sleep onset (WASO; min), the time spent in bed awake minus sleep onset latency. Actigraphy was used in conjunction with the EEG device to check the time between lights-out and lights-on, i.e., when the participant was lying down attempting to sleep. Both food intake and time of ingestion were recorded during the entire tour period. The meal plan included a variety of bread, grains, meat/fish, pasta/rice, fruit and vegetables to ensure the adequate intake of macro- and micronutrients.

### 2.3. Statistical Analyses

Simple descriptive statistics are reported as means ± standard deviations (SD). Comparisons between the baseline and nights throughout the tour were assessed through the difference in change scores. Effect size data (ES) was calculated to determine the magnitude of the change score and was assessed using the following criteria: <0.2 = trivial, 0.2–0.6 = small, 0.6–1.2 = moderate, 1.2–2.0 = large, and >2.0 = very large [14].

## 3. Results

### 3.1. Performance

**Figure 1:**
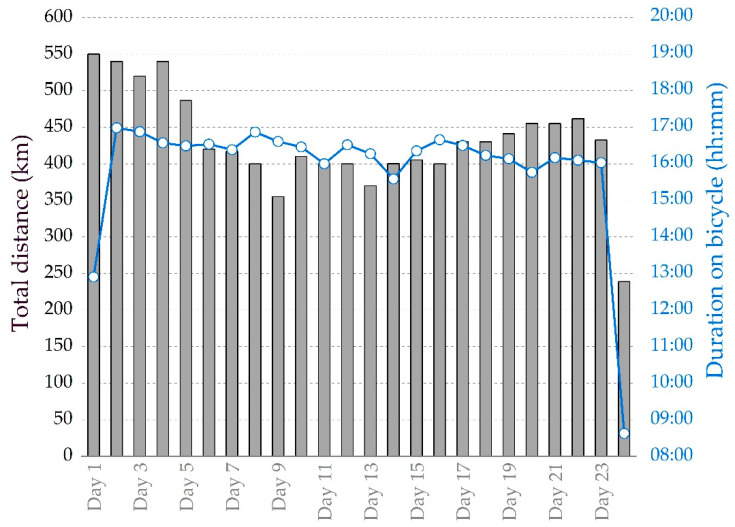
Progression of daily total distance and duration on bicycle over the duration of the 24-day tour.

The athlete completed 10,358.2 km in 24 days with a daily distance of 431.6 ± 67.2 km at an average speed of ≈27 km/h (Figure 1).

### 3.2. Sleep

**Figure 2:**
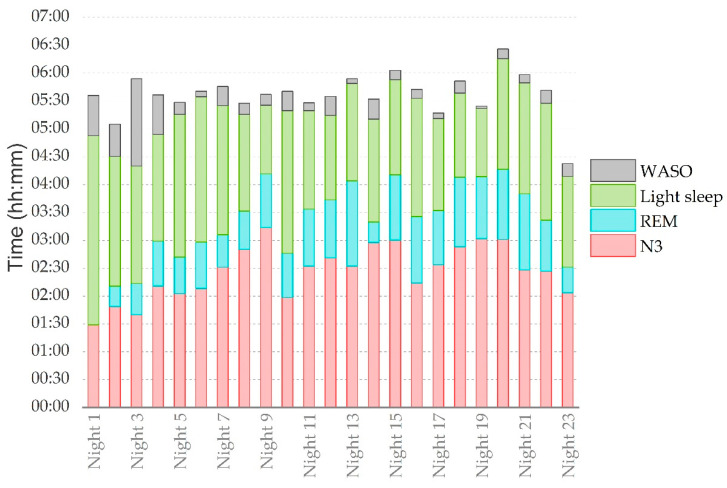
Progression of sleep stage distribution in the tour over the duration of 23 nights. WASO: wake after sleep onset; REM: rapid eye movement sleep; N3: slow-wave sleep.

The Horne and Ostberg questionnaire revealed that the subject’s chronotype was mid-range. Changes in the subject’s sleep behavior from the baseline were examined over the tour. Sleep onset latency throughout the tour (8 ± 8 min) was reduced compared to the measured sleep onset latency during the baseline (17 ± 18 min), with a moderate difference (ES = 0.7). Sleep duration throughout the tour (5:13 ± 0:30) was reduced compared to the baseline sleep duration (7:00 ± 1:00), with a very large difference (ES = 2.3). However, sleep efficiency (93 ± 6%) during the tour remained unchanged compared to the baseline sleep efficiency (93 ± 4%). The structure of sleep changed over time, with increasing proportions of N3 during the tour. The proportion of N3 during the tour (46 ± 7%) was compared to the measured N3 proportion during the baseline (27 ± 5%) and was found to be systematically outside the intra-individual variability (mean ± 1 SD), with a very large difference (ES = 3.1). The increase in the proportion of N3 occurred at the expense of REM, since the proportion of REM during the tour (16 ± 6%) was reduced compared to the measured REM proportion during the baseline (25 ± 5%), with a large difference (ES = 1.6). The distribution of sleep stages throughout the tour is presented in Figure 2.

Sleep onset latency, sleep duration, sleep efficiency, N3 proportion, and REM proportion reported during the two nights of recovery were 5 ± 0 min, 7:26 ± 0:29, 97 ± 0%, 32 ± 3%, and 20 ± 4%, respectively.

## 4. Discussion

The current case study allowed for an investigation of the sleep of an ultra-endurance cyclist engaged in a simulated 10,000 km tour. Here, the cyclist set a new world record, completing 10,358 km in 24 days with a mean daily distance of ≈432 km in approximately 16 h, i.e., an average speed of ≈27 km/h. Main results showed a ≈1 h 45 min reduction in sleep duration per night throughout the tour (05:13 ± 00:30), alongside a profound change in sleep structure over time with, notably, an increasing amount of slow-wave sleep (N3).

Previous studies have shown that sleep reductions of more than two hours per night alter sleep architecture. Slow-wave sleep, thus, acts as the primary drive mechanism of the sleep system [15]. In the present study, the chronic sleep reduction achieved, of ≈1 h 45 min per night during 23 consecutive nights, occurred alongside a sleep structure modification with very important proportions of N3 (range: 30–59%) observed during the tour. The two-process model of sleep regulation posits that a homeostatic process (Process S) interacts with a process controlled by the circadian pacemaker (Process C) [16]. While acknowledging that longer extreme sports events are associated with less neuromuscular fatigue than shorter events [17], it is very likely that the present daily important physical/mental load imposed on the subject maximized the sleep pressure. During slow-wave sleep, brain and core body temperature decrease, thus lowering energy utilization [18]. The subject’s chronotype was mid-range, with habitual preferential sleep timing between 23:30 and 7:00. During the tour, the subject kept a very regular sleep routine, with mean sleep start and sleep stop time—assessed by the EEG device—at 23:49 and 5:13, respectively. Therefore, according to the two-process model that successfully simulates the timing and intensity of sleep [16], all the conditions were met during the tour to maximize the proportion of N3. Accordingly, sleep onset latency throughout the tour was remarkably low (8 ± 8 min) and moderately reduced compared to the measured sleep onset latency during the baseline. This case study may be considered as a supplementary proof that regular physical activity improves the quality of sleep, as demonstrated elsewhere [19,20].

The effects of fragmented sleep on extreme endurance performance are very scant in the literature. Edinger et al. [21] studied two players involved in a week-long marathon tennis match under more severe sleep restricted conditions than the current study (4- to 5-h reductions per night). Results showed that the sleep restriction during the match resulted in a sleep stage distribution in favor of slow-wave sleep. Netzer et al. [1] performed a polysomnography continuously on a downhill skier during an 11-day performance. Results showed an average sleep time of ≈6 h per 24 h, similar to the sleep duration reported in the present study, but these sleep hours comprised a total of 36 min of N3 (11%) and 42 min of REM sleep (13%) [1]. Differences in EEG methodology and/or organization of sleep episodes (continuous night sleep vs. polyphasique sleep) and/or sleeping environment may explain the differences in sleep-stage distributions between studies.

Lahart et al. [3] studied competitors in The Race across America, a solo-bicycle race in the USA, which imposes a toll on physical and mental resources, similar to those encountered in the present study. Results demonstrated that cyclists attained an almost threefold increase in sleep duration and higher sleep efficiency during 6 h rest periods compared to 3 h rest periods. Therefore, the balance between average cycling velocity and sleep time during a race must be optimized [3]. According to Bianchi et al. [22], it would appear that ultra-endurance athletes involved in short events might consider sleep as an impediment to achieving their race goals and will forgo sleep for a competitive advantage, i.e., sleep time is lost time; whereas long events, such as multiday events, may present a situation in which sleep cannot be dismissed and, thus, becomes a key factor in race completion. In the present study, sleep efficiency (93 ± 6%) during the tour remained rather constant, which may be notably explained by the sleeping strategy retained a priori by the subject (i.e., a continuous night sleep during habitual preferential sleep timing). Future studies are required to ascertain the potential interest of sleeping strategies (e.g., multiple napping) [23,24] aimed at maximizing the balance between cycling performance and sleep quantity/quality during a multiday ultra-endurance event. Additionally, future investigation of varied sleep strategies should also consider the interest of pre-planned sleep strategies [25]. In the previously mentioned study [21], one of the tennis players incurred a slow-wave sleep debt during the marathon match, which was paid back during recovery. In the present study, sleep duration (7:26 ± 0:29) and N3 proportion (32 ± 3%) remained slightly higher during the two nights of recovery compared with the baseline values, which appears to be consistent with previous laboratory studies documenting the priority of the slow-wave sleep drive.

Lastly, some methodological aspects of the present case study must be considered. We cannot exclude a potential bias arising from repeated measures on the same subject. This contributes to the low level of evidence assigned to non-analytic studies, such as case reports and case series [26]. Caution should be taken in generalizing these findings beyond the current athlete. However, the successful implementation of the reduced-montage dry-EEG device throughout the tour positively contributes to the ultra-endurance literature, by expanding upon the very limited knowledge on sleep architecture modification during such events. Progression of sleep research in this direction will help move more towards guidance that competitors can directly apply.

## 5. Conclusions

The current ultra-endurance event reduced sleep quantity, besides a notable modification in sleep architecture. The increase in slow-wave sleep during the race may be linked to the role of slow-wave sleep in physiological recovery. Although fragmented sleep is portrayed negatively, this case study was conducted as an example applied to the real world: athletes with restricted sleep can maintain physical performance during a 24-day ultra-endurance tour.

## Data Availability

Data will be shared upon specific request.
